# Supplementary material for: Influence of the Hypoxia-Activated Prodrug Evofosfamide (TH-302) on Glycolytic Metabolism of Canine Glioma: A Potential Improvement in Cancer Metabolism
Source: Cancers (Basel). 2023 Nov 22;15(23):5537. doi: 10.3390/cancers15235537 (PMC10705110; doi:10.3390/cancers15235537)
Supplement: Supplementary file 1 [file cancers-15-05537-s001.zip › cancers-2627712-supplementary.pdf]

Supplemental data

Immunohistochemistry

Protein expression of HIF-1α and PDK1 were evaluated by immunohistochemical analyses (IHC) using paraffin-embedded tissues. Sections of the collected tumor samples embedded in paraffin wax blocks were prepared on slides. Samples were subjected to a dewaxing and hydration process by soaking in baths ranging from xylene to graded alcohol, and then washed in PBS (pH 7.2). Next, the slides were fixed in methanol for 15 s at room temperature and then air-dried. The slides were then immersed in 0.3% H_2O_2 in methanol for 20 min at room temperature to inhibit endogenous peroxidases, and then washed once more in PBS. Blocking with 10% normal goat serum (Histofine SAB-PO Kits; Nichirei Bioscience, Tokyo, Japan) was then carried out for 10 min, after which the slides were washed three times in PBS. The samples were incubated overnight at 4°C with primary rabbit polyclonal anti-HIF-1α (NB100-134, Novus Biologicals, CO, USA) and anti-PDK1 (ADI-KAP-PK112-F, Enzo Life Sciences NY, USA) diluted to 1:1000 in PBS, washed three times with PBS, and then incubated with secondary goat anti-rabbit IgG (Nichirei Bioscience, Tokyo, Japan) diluted 1:3 in PBS for 30 min at 4°C. After three more washes with PBS, the samples were incubated in peroxidase streptavidin (Nichirei Bioscience, Tokyo, Japan) for 30 min at 4°C, washed three times with PBS, and then visualized after staining with 3,3′-diaminobenzidine (Sigma-Aldrich, Sigma, St. Louis, Missouri) and hematoxylin. All HIF-1α positive cells in IHC were classified as tumor cells in which the nucleus and/or cytoplasm were distinctively stained. Ten high-power fields (HPFs) were selected in sites containing > 50% tumor cells, and the number of HIF-1α- and PDK1-positive cells for each site was accurately counted using a digital microscope (Biorevo BZ-9000; Keyence) and image analysis software (IM50 image manager version 1.2, Leica). Finally, the median of the HIF-1α- and PDK1-positive cells was determined, the dogs were divided into 2 groups of high HIF-1α expression (> median of HIF-1α-positive cells) and low HIF-1α expression (< median of HIF-1α-positive cells), and the

correlation coefficient between rate of HIF-1α and PDK1 positive cells in GL dogs was determined. Data were presented as the mean of triplicate samples, and the experiment was repeated three times independently.

Enzyme-linked immunosorbent assay (ELISA)

After the cells were cultured for 24 h under normoxia (21% O_2) and hypoxia (1% O_2), the whole, nuclear and cytoplasmic protein expression of HIF-1α were evaluated by Enzyme-linked immunosorbent assay (ELISA). The nuclear and cytoplasmic proteins were extracted from them with the Nuclear/Cytosolic Fraction Kit (Cell Biolabs, San Diego, CA, USA), according to the manufacturer's instructions. Briefly, the cells were homogenized in ice-cold cytosol extraction buffer and cell lysis reagent was added. The homogenate was centrifuged at 800 × g, and the supernatant was separated and saved as the cytosolic fraction. The nuclear fraction was washed several times with nuclear extraction buffer and incubated on ice for 30 min. The mixture was centrifuged at 14,000 × g for 30 min. The supernatant was separated and stored at −80 °C until analysis. The ELISA protocols were applied according to the manufacturer's instructions for Canine HIF-1α ELISA Kit (EK8714, MD, USA). Briefly, the diluted antibodies were added into wells of a 96-well ELISA plate. The plate was sealed to prevent evaporation and incubated for 18 h at 4 °C to immobilize the antibody. The diluted antibody was removed and the plate washed with washing solution. Blocking buffer was added to each well and incubated for 1 h at 37 °C to reduce non-specific binding of the target protein to the well. Blocking buffer was removed and the plate washed with the washing solution. Samples were diluted with sample dilution buffer and 100 μL of each sample was added to each well. For the calibration curve, a dilution series of the standard was prepared on the same plate. The plate was incubated for 1 h at 37 °C, samples and standards were removed and the plate washed with washing solution. The detection antibody was diluted in sample dilution buffer and 100 μL added to each well, then incubated for 1 h at 37 °C. After the reaction, the detection antibody was removed and the plate washed with the washing solution. Enzyme-labeled secondary antibody was diluted with sample dilution

buffer and 100 μL was added to each well the incubated for 1 h at 37 °C. After the reaction, the secondary antibody was removed and the plate washed with washing solution. A substrate solution was added and allowed to incubate until the color developed. When the color has been developed sufficiently, a stop solution was added to stop the reaction. Then, the absorption was measured at 450 nm with an iMark microplate spectrophotometer (Bio-Rad, Hercules, CA, USA). Data were presented as the mean of triplicate samples, and the experiment was repeated three times independently.

Western blotting

After the cells were cultured for 24 h under normoxia (21% O_2) and hypoxia (1% O_2), the whole protein expression of GLUT1 and PDK1 were evaluated by western blotting analysis. The cells were washed twice with ice-cold Tris-buffered saline (TBS; Sigma) and then lysed with 100 μL of radio-immunoprecipitation assay (RIPA) buffer (Sigma). The protein concentrations were determined with the Bradford protein assay using bovine serum albumin (BSA; Sigma) as the standard, and 20 μg of protein from each sample was loaded onto a gel for electrophoresis. The proteins were denatured, subjected to sodium dodecyl sulfate polyacrylamide gel electrophoresis (10% gels; Sigma), and electrotransferred onto nitrocellulose membranes (Whatman, Piscataway, NJ, USA) in a semidry transfer apparatus (Bio Craft, Tokyo, Japan). The membranes were incubated for 1 h at room temperature in a blocking solution: 10 mM Tris-HCl (pH 7.4), 0.15 M NaCl, 0.1% Tween 20, 1% BSA, and 0.05% NaN_3. The membranes were then incubated overnight at 4 °C with the primary antibodies, including a rabbit monoclonal anti-GLUT1 (EPR3915, diluted 1:1000; Abcam, Cambridge, UK), anti-PDK1 (ADI-KAP-PK112-F, diluted 1:1000; Enzo Life Sciences NY, USA) and a mouse monoclonal antibody directed against β-actin (G043, diluted 1:1000; Abcam, Cambridge, MA, USA). The membranes were washed three times for 5 min each with Tween-TBS (10 mM Tris-HCl, 0.15 M NaCl, and 0.1% Tween 20) and then incubated with a horseradish-peroxidase-conjugated anti-rabbit-IgG secondary antibody (diluted 1:1,000; Fischer Scientific

Thermo, Pittsburgh, PA, USA) in Tween-TBS for 1 h at room temperature. The immunoreactive bands were visualized with a chemiluminescence system (Ez-Capture MG, Atto, Tokyo, Japan), using Amersham ECL Prime Western Blotting Detection Reagent (GE Healthcare, Princeton, NJ, USA). The bands of GLUT1 and PDK1 on the western blots were quantified with the ImageJ software (version 1.451: http://imagej.nih.gov/ij/) and are presented as relative intensities normalized to that of β-actin. Data were presented as the mean of triplicate samples, and the experiment was repeated three times independently.

Flow cytometry

The cells were treated for 24 h with the IC50s of TMZ and EVO after culture for 7 days under normoxia (21% O_2) and hypoxia (1% O_2), then apoptosis was analyzed using flow cytometry. The rate of apoptosis was further determined using an annexin V-FITC apoptosis detection kit (Blue Heron Biotechnology, Bothell, WA, USA). Briefly, 1×10^6 cells were harvested and washed with cold PBS and then resuspended in 1 ml of 1× binding buffer. After incubation with annexin V-FITC and PI, the apoptotic cells were detected by flow cytometry within 1 hr. The upper left (UL) quadrant ((AnnexinV-FITC)−/PI+) denotes necrosis, the upper right quadrant (AnnexinV+FITC)+/PI+) denotes late apoptosis, the lower right (LR) quadrant ((AnnexinV- FITC)+/PI−) denotes early apoptosis and the lower left (LL) quadrant ((AnnexinV-FITC)−/PI−) denotes viable cells. Total apoptosis was equal to the sum of the late and early apoptosis values. Data were presented as the mean of triplicate samples, and the experiment was repeated three times independently.

Metabolome analysis

Metabolome analysis was performed using tumor tissue sections from three individual murine models, which were xenografted into tumor cells (G06A, J3TBg and SDT3G, respectively) cultured under normoxia and hypoxia. Briefly, the number and weight of cells in conventional two-dimension culture were measured in advance, and the number of cells in the tumor tissue sections was calculated based on the weight. The tumor

tissue sections might contain stromal cells, excluding tumor cells; however, since the number of tumor cells was significantly higher than the number of stromal cells in the tissue sections, the amount was estimated to be within a negligible range. Metabolome measurements were performed at Human Metabolome Technologies, Inc. (Tsuruoka, Yamagata, Japan). Briefly, frozen samples were plunged into 50% acetonitrile/Milli-Q water with internal standards (H3304-1002; Human Metabolome Technologies) at 0 °C to inactivate enzymes. The tumor tissue sections were homogenized three times at 1500 rpm for 120 s using a tissue homogenizer (Microsmash MS100R, Tomy Digital Biology), and the homogenate was centrifuged at 2300 g and 4 °C for 5 min. Subsequently, 800 µL of the upper aqueous layer was subjected to centrifugal filtration through a Millipore 5-kDa cut-off filter at 9100 g and 4 °C for 120 min to remove proteins. The filtrate was concentrated in a centrifuge and resuspended in 50 µL Milli-Q water for capillary electrophoresis-mass spectrometry (CE-MS) analysis. We analyzed >1000 metabolites, especially water-soluble and ionic metabolites related to central energy metabolism.

Figure S1

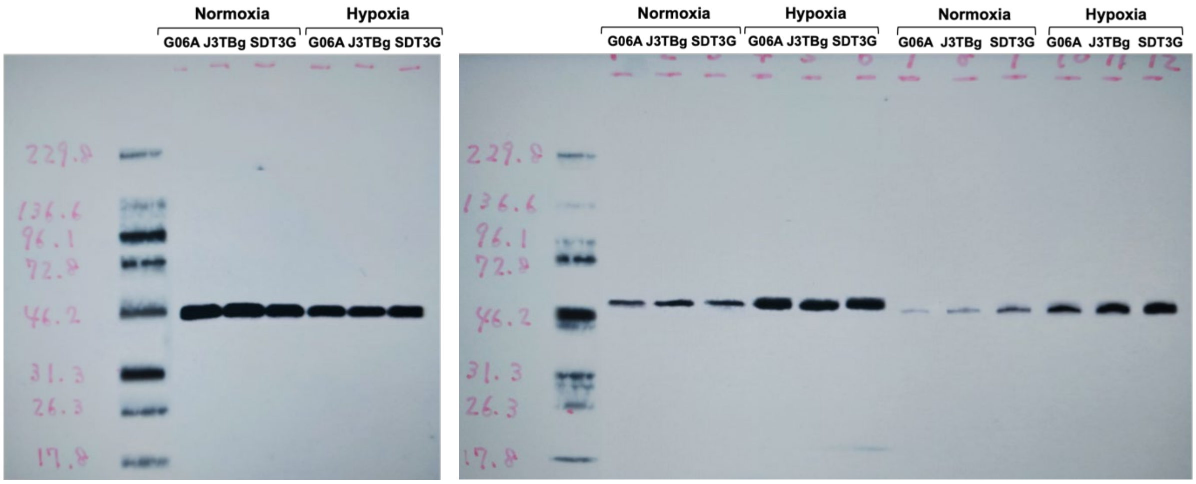

Whole proteins of β-actin (left figure), GLUT1 and PDK1 (right figure) in G06A, J3TBg, and SDT3G cells after 24 h of normoxic or hypoxic culture. Immunopositive bands are represented as relative value (%) normalized to β-actin.
